# A Comparative View of the Principal Theories Which Have Prevailed in Chemistry, &c.

**Published:** 1799-06

**Authors:** 

**Affiliations:** Sen. of Vienna


### *A Comparative View of the principal Theories which have prevailed in Chemistry, &c. By Dr.* Frank, *Sen. of Vienna.*

[*Concluded from Numb.* III. *pp.* 269—273.]

WHEN we attentively consider the phenomena which natural bodies exhibit to our senses; when we compare the relative conditions in which these bodies are placed in respect to others, we are enabled to abstract certain rules or principles, by which we can ascertain the relation of such bodies, before they are exposed to causes which will produce various effects on them; or, in other words, from the reciprocal affinity subsisting between bodies, we are able to determine their momentary relation to others, whether known or unknown. We know, for instance, from observations and experiments accurately made, that all inflammable bodies assume the state of combustion, and remain in it only as long as they are in contact with oxygen, in different degrees of temperature: hence we are led to conclude, that in every instance where inflammable bodies are placed in contact with oxygen, and in the requisite temperature, combustion will necessarily take place; and, on a similar foundation, we predicate in every process of combustion, that an inflammable body and oxygen are the two active agents.

Such a law or principle, therefore, as is derived from pure experience, and is calculated to explain a particular phenomenon in the material world, is called a *theory*; while the aggregate of such principles as enable us to explain a number of phenomena, or a whole series of events, is termed a *system*.

The philosophic inquirer will, however, soon remark, that no experimental pursuits possess mathematical certainty; that the criterion of *certainty* cannot be discovered in the explanation of all the phenomena of nature; and that we must be satisfied, in almost every instance, with greater or less *probability*.

However discouraging this circumstance may be to the progress of the human mind in scientific researches, it is, at the same time, attended with beneficial consequences: for it is this imperfection in man, considered as a reflecting being, that urges him to investigate with so much zeal, the causes of the phenomena taking place, whether in the circle of animate or inanimate nature; and to trace those hidden or manifest powers, by which she produces the endless variety of physical effects.

As long as these causes are discoverable by the reasoning faculty of man, he will not readily deviate from the true path of inquiry; as he is capable of avoiding whatever may divert his attention, or disturb him in the perception of such phenomena.

But we are not, in every instance, equally fortunate: these causes frequently vanish from our researches; sometimes they are covered by an impeneimpenetrable veil, and removed from the perception of the most sagacious inquirer. When the capacity and application of the external organs are suspended, our perceptive faculty is likewise arrested: thus the indefinite characters vanish from the circle of sensible qualities; and not being able to ascertain the necessary condition of our intuitive knowledge, *i. e.* that of *space* and *time*, we are at once checked in our search after certainty. Nevertheless, man is ever anxious to indulge in such inquiries, as he is extremely averse to leaving off, where the senses resign their office to the understanding. His intellectual powers revolt at such fetters, and, venturing into the regions of fancy, an immense field of ideas presents itself to his view; in the developement of which, he finds ample scope for the exercise of his mental faculties. He compares, combines, and arranges his ideas, being principally guided by *analogy*. He adopts this as the scale by which he measures the relative truth of his notions; it serves him as a surrogate of perception, and is his faithful companion in every step. But, if he meet with obstacles, and this resource fail him, he is obliged, as it were, to build a bridge, and to substitute an *hypothesis*, in order to cover a chasm which he cannot, without reluctance, suffer to remain entirely open.

As we cannot, in the immense field of physics, always attain positive knowledge, we are very frequently obliged to be satisfied with opinions and conjectures: if these be accompanied with plausible arguments, our satisfaction will be more or less complete. The highest degree of probability, and complete applicability, therefore, are the characters of a well-founded hypothesis—it ought to be perfectly consistent with nature; its principles should be simple, and of universal application.

Let this introduction suffice to explain the point of view from which the theories of every experimental science, and consequently those of chemistry, ought to be considered by the inquisitive student. It is necessary that he should previously settle that point, in order to prevent him either from raising the theory too high, or degrading it more than it actually deserves. Facts ever remain unaltered; but the manner of explaining them may be different. An accurate estimation of the methods adopted to account for the phenomena of nature, and a cool comparison with the facts on which they rest, will soon enable us to discover whether the one or the other of these methods deserve the preference; so that neither length of time, nor the authority of names, nor the country of the founder of a particular theory, ought to influence our determination. Such distinctions are neither rational nor scientific; for in the pursuits of science, we should attend to facts only, without paying regard to their origin, or who discovered them, except so far as they serve to illustrate literary history.

If theſe principles be applied to the different chemical ſyſtems, the outlines of which we have exhibited in the former part of this eſſay, we may venture to examine them more minutely, as we have before given only a general account of the principles on which they are founded.

The doctrine firſt propoſed by Stahl is, indeed, pregnant with too many imperfections to deſerve general approbation. Phlogiſton is the centre round which the whole ſyſtem turns; although in the ſenſe in which Stahl and his adherents conceived the nature of that ſubſtance, together with the properties which they attributed to it, it is, in fact, a non-entity. In former times, when chemiſtry was in its infancy, people were eaſily ſatisfied with ſuch explanations: but ſince the objects of chemiſtry have been multiplied; ſince numberleſs experiments and diſcoveries have been ſucceſsfully made, and ſince the diverſified phenomena of nature have been more critically inveſtigated, the imperfections of the Stahlian ſyſtem have been detected, eſpecially ſince chemiſts have been fortunate enough to trace the origin of the gaſeous fluids. In conſequence of theſe improvements, modern chemiſts ſoon diſcovered the many paradoxical aſſertions contained in Stahl's ſyſtem; and endeavoured to improve and adapt it ſo as to render it, in ſome degree, applicable to later theories: and although they did not fail to transform it with art and ingenuity, ſo that it no longer reſembled its priſtine form, it nevertheleſs remained too unwieldy, and too little conſonant with nature, to claim any permanent eſtabliſhment.

The phlogiſton of Stahl was maintained to be an elementary combination of earth and fire, incapable of penetrating through veſſels. It was affirmed, that this matter formed all combuſtible ſubſtances; produced light and heat in combuſtion, by being decompoſed into its conſtituent parts; and laſtly, that phlogiſton was the baſis of all metals. It was, however, found out in later times, that combuſtion could only take place when vital air (oxygen) had acceſs to that proceſs; it was clearly perceived, that this aëriform ſubſtance was diminiſhed, and underwent a very obvious change of its nature. The chemiſts of the old ſchool endeavoured to explain this phenomenon, by admitting that vital air is a ſubſtance deprived of all phlogiſton, which ſubſtance had a greater affinity to phlogiſton, and conſequently abſtracted it from bodies ſubjected to combuſtion. But, according to Stahl's idea, phlogiſton ſhould have been decompoſed; fire ſhould have eſcaped in the effect of ſenſible heat and light, and left behind the baſe of earth. Of this laſt, however, not the leaſt trace could be diſcovered; on the contrary, it was obſerved, that bodies, notwithſtanding the eſcape of phlogiſton, had actually increaſed in weight during combuſtion.

These

Thefe difficulties were infurmountable; they induced Dr. Crawford to maintain that vital air contained a great proportion of fpecific, or fixed elementary fire, but that it parted with this, as foon as it met with an opportunity of exchanging it for phlogifton, whereby it was changed into phlogiftic air: fuch was the cafe in the procefs of combuftion. But it was likewife inconfiftent with experience; that in the combuftion of phofphorus all vital air difappeared; and that no elaftic fluid which could enter into combination with phlogifton, remained behind: befides, the aëriform refiduum in the combuftion carried on in pure vital air, was, in moft inftances, an aërial acid which combined with cauftic lime and vegetable alkali, and had nothing of what was called phlogiftic air. From whence did this arife? It was indeed maintained, that it had been prefent in the fubftance previous to its combuftion, and was only difengaged during that procefs. But on the other hand it was found, that this aërial acid, together with the refiduum of the body fubjected to combuftion, exceeded the former weight of that body.—In order to explain this, fome natural philofophers have maintained, that phlogifton is a fubftance of negative fpecific gravity, or fuch as renders bodies fpecifically lighter, and that its point of attraction muft be in that univerfal luminary the fun: again, others believed that vital air is virtually water, expanded by the matter of heat (caloric), which water is depofited by vital air, and imparted to bodies during combuftion; a circumftance by which they endeavoured to account for the increafe of weight thus occafioned.

The doctrine of Lavoisier, which exploded the exiftence of phlogifton, at length appeared, and excited univerfal attention. Former opinions were controverted, and phlogifton banifhed to the regions of fancy, becaufe it could no where be exhibited to the fenfes; for, though it fhould never be incoercible, it could in no inftance be difcovered where it fhould have been feparated. Thus calcined mercury was treated in clofe veffels, without the addition of a phlogiftic body, and neverthelefs was reduced to its metallic form. Whence, then, did the phlogifton proceed, and how had it combined with the calx of the metal? It was afferted that phlogifton was incoercible, and virtually fire, in a fixed ftate. But even granting this hypothefis, there arifes another important queftion: why could the metal not be calcined without coming in contact with pure air, as it was not prevented from depofiting its fixed fire?

In this manner the doctrine of phlogifton was involved in ftill greater difficulties, by which the way was paved to the fyftem of chemiftry propofed by the ingenious Lavoifier. He fupported his opinions by accurate and repeated experiments, the refult of which did not much vary, when repeated by other chemifts, in different countries. Such was particularly the caſe with the leading opinion, that vital air depoſited or imparted its elementary baſe to combuſtible bodies, increaſed their weight, and changed their form; this baſe, on account of its property of forming acids, in combination with many other bodies, he denominated oxygen. Similar proofs of the truth of Lavoiſier's aſſertions were given in the generation of water, as well as in demonſtrating other principles of the antiphlogiſtic ſyſtem.

Thoſe who do not acknowledge the ſuperior advantages of this ſyſtem, are actuated either by prejudice in favour of former ſyſtems, or injuſtice to the merits of the French. It is incomparably more perfect than that of Stahl, and is indiſputably better calculated to afford a complete and ſyſtematic theory of chemiſtry. The moſt eſſential particulars relative to the compoſition and decompoſition of bodies can be ſatisfactorily explained, and the different phenomena accounted for upon fixed and determinate principles. Let us conſider the important influence this ſyſtem has produced on ſeveral of the practical ſciences, which are more or leſs connected with chemiſtry. Phyſiology, pathology, the practice of medicine, as well as agriculture, have each of them derived conſiderable advantages from the illuſtrations of the antiphlogiſtic ſchool. We may reaſonably expect to be ſtill more benefited by the reſearches of chemiſts, if they continue their efforts for the improvement of theſe ſciences, and if phyſicians will devote their attention, more than they have hitherto done, in applying the principles of chemiſtry to the healthy as well as the diſeaſed ſtate of the animal body.

I ſhall illuſtrate this remark by a ſhort digreſſion.—The author of the criticiſms on the theories of Dr. BEDDOES *, as well as the great phyſician Hufeland, very juſtly obſerve, that in applying chemical principles to the elucidation of medical ſcience, due attention ought always to be paid to the preſence and activity of vital power in the animal body; a power which muſt neceſſarily occaſion conſiderable deviations: and I here repeat, that this remark is well founded, as many modern practitioners have occaſionally gone to ſuch lengths, that the cooleſt obſerver who takes the trouble to examine ſome of their theories, cannot refrain from ſmiles; for inſtance, at the manner in which they explain muſcular action. But it would be unjuſt to impute theſe errors to chemical ſcience, as they proceed only from falſe theory, and an extravagant fondneſs in its application.

Satisfactory, however, as the ſyſtem of Lavoiſier appears in many points, it is, nevertheleſs, if examined without prejudice, not altogether free from errors

* Journal of Inventions, No. IX. to be extracted in a future Number.

errors and imperfections. Its opponents particularly object, that the baſe of vital air does not deſerve the title of oxygen, as many combinations of it are far from being acids. This, indeed, cannot be denied, yet it is alſo true, that all acids hitherto known, originate from that ſource; nay, that ſome metallic calces, by a ſuperabundant communication of vital air, are changed into the ſtate of an acid; and hence it may not be improper to borrow the name of oxygen, or the acidifying principle, from that property. But if the objection be ſtill maintained, I conſider it of little importance, as the point of diſpute relates only to a term, and as terms ſerve only to repreſent the ſigns by which we render our ideas intelligible to others. The ſign itſelf, however, has no eſſential influence on the object ſignified, although it be true that each bears a cloſe relation to the other, inaſmuch as the character of the latter ought to be expreſſed, in the cleareſt poſſible manner, by means of the former. If, therefore, a more appropriate term could be contrived, to indicate the baſe of vital air, it would no doubt be adopted by every rational chemiſt, in preference to that of oxygen; but until this be done, we may be permitted to retain that term as the moſt ſuitable to the purpoſe.

The ſtrictures which have been made by others, that this oxygen cannot be exhibited in a perfectly pure ſtate, are equally unimportant. There is no ſubſtance in nature that exiſts without being combined with caloric; and how is it poſſible to exhibit a body which, in this combination, is changed into a gaſeous ſtate, in any other form than that of gas?

But a more important objection againſt the ſtability of the antiphlogiſtic ſyſtem has been frequently ſtarted by its opponents; it relates to the defective explanation of light, and the phenomena reſulting from that element. Lavoiſier has repreſented light and heat as mere modifications of one and the ſame ſubſtance; an aſſertion which certainly cannot be maintained. Although we were to admit that light, heat, electric, nay, even magnetic matter, are modified ſubſtances, yet they are ſuch only in the hands of *nature*, and not in thoſe of the chemiſt. All theſe ſubſtances preſent themſelves to the obſerver under ſo diverſified an aſpect, and are poſſeſſed of properties ſo peculiar, that we are neceſſarily induced to conſider each of them as ſelf-ſubſiſtent, and conſequently to pay no attention to their *poſſible* modifications. In this reſpect we meet with an eſſential deficiency in the ſyſtem of Lavoiſier, who maintains that the phenomena of light and heat are only the reſults of that *condition*, under which any particular ſubſtance is diſengaged.

This inconvenience, or rather incongruity, was indeed ſoon diſcovered by ſome acute chemiſts. They admitted the preſence of two different ſubſtances in in theſe phenomena, and aſſerted that both light and heat were united to the baſe of vital air; and that, in the proceſs of decompoſition, they were either ſeparated in a viſible form, or entered into new combinations. Many phenomena appeared to confirm the truth of this aſſertion: thus, the perfectly pellucid nitric acid again acquires its colour, and emits red fumes, when expoſed to the rays of the ſun, an effect which heat alone does not produce; thus alſo, the ſuperſaturated muriatic acid is, under ſimilar circumſtances, reduced to the ſtandard of common muriatic acid; and thus, laſtly, plants emit vital air, only when expoſed to light, but never in a contrary ſtate. In all theſe inſtances, vital air is formed, while the elementary baſe, abundantly contained in the acids before-mentioned, on account of greater affinity, fixes light and caloric, and aſſumes the gaſeous form; but by theſe means, the acids themſelves are reduced to the ſtate of imperfect oxydation. In plants, the oxygen is ſeparated from the hydrogen, with which it forms water, as ſoon as the eſſential conditions (that is, light and heat) be preſent, in which only it can be changed into vital air.

Theſe phenomena ſufficiently prove, that the theory which ſuppoſes the matter of light to paſs into oxygenous gas, is far from being a vague hypotheſis, while they bear ſtrong evidence of the actual preſence of that ſubſtance in the gas. If this propoſition be once admitted, it will not be difficult to explain all the phenomena of combuſtion, upon the principles of the antiphlogiſtic ſyſtem.

There can be only one opinion among chemiſts, that oxygen is indiſpenſably neceſſary in every proceſs of true combuſtion; but we ſhould meet with inſurmountable difficulties in explaining the phenomena of radiant heat, of lightning, or of mere heat, if, in oxygen gas, thoſe ſubſtances were contained which, in a diſengaged ſtate, produced theſe phenomena, accordingly as they operate, individually or united.

By this modification, therefore, the apparent inconſiſtency of the ſyſtem of Lavoiſier may be eaſily reconciled, and all objections effectually obviated. It further adds to the ſatisfaction the philoſophic inquirer derives from comparative reſearches, that the modification before detailed, in its principal points, agrees in a ſtriking manner with the ſyſtem of the two German chemiſts, Gren and Richter.

The chief point on which the latter ſyſtem reſts, and by which it appears to be diſtinguiſhed from the antiphlogiſtic, is the doctrine reſpecting the exiſtence of the matter of light in combuſtible bodies. But, upon attentively conſidering the ſyſtem of the French philoſopher, we ſhall find, that the difference is neither eſſential nor founded in reality.

The

The two chemiſts before-mentioned were, in fact, the firſt who repreſented the doctrine of this ſyſtem relative to the matter of light, in its full extent; a ſubject which the antiphlogiſtians themſelves had hitherto left undetermined.

The matter of light, however, as well as that of heat, is an incoercible ſubſtance, which, being diffuſed through the univerſe, is, like every other ſubſtance, ſubject to the laws of chemical affinity; conſequently, this matter alſo, like that of heat, is capable of entering into combinations with all ſubſtances exiſting in nature. Is it, therefore, not perfectly conſiſtent that theſe natural bodies, as ſoon as there ſubſiſts between them and oxygen a ſtronger degree of affinity than between them and the matter of light, ſhould enter into combination with the former; and that if the product ariſing from this combination manifeſt either a very ſlight degree of, or no attraction at all, for the latter (that is, when the body, combining with oxygen is of a combuſtible nature), they part, under certain conditions, with *their* matter of light, which in this caſe eſcapes, together with *that* of the oxygen, either alone, or conjointly with its caloric, becomes manifeſt to the ſenſes, or forms new combinations. Thus ariſe the different degrees of combuſtion, both in point of violence and duration, accordingly as the quantity and modification of the incoercible ſubſtances (the matters of light and heat), diſengaged from oxygen, as well as from the combuſtible body, are different in their nature.

This is a pure principle of the antiphlogiſtic ſyſtem, although it has not been hitherto expreſſed in that form by the French chemiſts. The idea which, in their ſyſtem, is connected with the term *lumiere* (matter of light), and the parallel in which this laſt ſtands with the matter of heat, is an inconteſtible proof of the preceding aſſertion. I believe that no friend of that ſyſtem will be ſo groſsly inconſiſtent, as to deviate from this explanation, or to maintain, that oxygen is the *only* ſubſtance capable of fixing and receiving light.

The changes which ſeveral ſubſtances undergo, when expoſed to light, as for inſtance, the muriat of ſilver, the ſolution of iron in vitriolic ether, &c. are ſufficient evidence of its influence and fixation. But ſtill more concluſive, in this reſpect, are the experiments which have lately been made by the Dutch chemiſts, and which deſerve every attention, as the facts thereby eſtabliſhed have long been known, although little attention has hitherto been paid to them. Theſe intereſting experiments relate to the generation of flame, in mixing melted ſulphur with metals. This flame which, ſo far from being the reſult of true combuſtion, is, according to the more appropriate term of Van Mons, rather that of ignition, or, as I would expreſs it, of inflammation, very probably ariſes from the circumſtance, that a part of the ſpecific matter matter of light contained in ſulphur and the metals, is ſeparated; becauſe the union thence ariſing, or the ſulphurated metal, poſſeſſes a leſs capacity for this elementary matter than both ſubſtances, jointly conſtituting the product, poſſeſs in a ſeparate ſtate.

That oxygen can have no ſhare in that inflammation is obvious, becauſe this phenomenon not only takes place in other ſpecies of gas, incapable of ſupporting the combuſtion of bodies, ſuch as the hydrogen, and carbonic acid gas, but likewiſe in a vacuum; beſides which, the preceding aſſertion is principally ſupported by the fact, that the combination thence reſulting is not a ſulphureo-acid, but a ſulphurated metal.

Dr. Richter, indeed, explains this phenomenon in a different manner, and accounts for it by the decompoſition of a ſmall portion of water, adhering to ſulphur (be it ever ſo well dried); a conjecture to which he is led by obſerving, that a ſlight trace of ſulphuric acid is generated in the proceſs. It does not, however, appear that the ſmall quantity of oxygen diſengaged ſufficiently accounts for the origin of the phenomenon, inaſmuch as this element, though contributing its ſhare to produce the phenomenon, ſeems to be altogether ineſſential.

If, therefore, we admit the preſence of the matter of light in all natural bodies, if it be granted, which cannot, indeed, be denied, that this ſubſtance is more or leſs, either ſingly, or combined with others, diſengaged in the chemical change of bodies, and thus produces the phenomenon of light, we are at once enabled to explain, in a conſiſtent manner, and with a degree of probability, all the phenomena in which light has any ſhare.

Whether we adopt the idea of Dr. Richter, that light is a compound body, or whether we believe with Profeſſor Goettling, that light is a ſimple element in a free ſtate, is in my opinion of no importance. Both conjectures admit of unequivocal proofs, and both explain the phenomena in an eaſy and ſatisfactory manner.

Notwithſtanding theſe conceſſions, we cannot pay implicit reſpect to the different theorems laid down in the ſyſtem of Profeſſor Goettling, as they are liable to many material objections, which have been already made to them by ſeveral chemiſts. The principal points in which this ſyſtem differs from the antiphlogiſtic ſyſtem of the French, or that of Richter, the non-exiſtence of azote, and the opinion that the baſe of nitre, or azotic gas, is a combination of oxygen and azote, have not been at all proved.

The experiments which induced Goettling to maintain this opinion, have been frequently repeated by Iselin, Lind, Jaeger, and Scherer, but have not proved in any degree ſatisfactory.

Scherer in particular has, by a repetition of theſe experiments, and the addition of ſeveral others, given the moſt deciſive proofs of his ſkill in chemiſtry, and afforded the greateſt accuracy of which the ſcience is capable: he has proved that phoſphorus neither emits light in *pure* azotic gas, nor is changed into an acid, but that theſe phenomena happen only when the azotic gas employed in the experiment, contains a portion of oxygen: for, when he procured azotic gas by expoſing phoſphorus in atmoſpheric air to the influence of red-hot coals, for the ſpace of an hour, he obſerved that the phoſphorus incorporated with the air, remained unchanged, and that the emiſſion of light did not take place. Mr. Goettling indeed objected, that the azotic gas thus prepared, contained phoſphorus in ſolution, and was thereby prevented from decompoſing the phoſphorus incorporated with it. This objection, however, is of no weight, if we admit the principle, that the greater the ſurface of the body is which is to be decompoſed, the eaſier will the body, which is to decompoſe it, operate.

At preſent, therefore, the theory of Goettling is not firmly eſtabliſhed, many important points ſtill remaining in doubt. Whether theſe doubts will ever be removed, time muſt determine: till they are, we may be permitted to adhere to that ſyſtem which appears moſt conſiſtent and ſatisfactory.

G. R. Frank.

